# Study on the Nanomechanical and Nanotribological Behaviors of PEEK and CFRPEEK for Biomedical Applications

**DOI:** 10.3390/polym10020142

**Published:** 2018-02-02

**Authors:** Jian Song, Hongyu Shi, Zhenhua Liao, Song Wang, Yuhong Liu, Weiqiang Liu, Zhongxiao Peng

**Affiliations:** 1 State Key Laboratory of Tribology, Tsinghua University, Beijing 100084, China; songj13@mails.tsinghua.edu.cn (J.S.); shihongyu1991@126.com (H.S.); 2 Biomechanics and Biotechnology Lab, Research Institute of Tsinghua University in Shenzhen, Shenzhen 518057, China; liaozh@tsinghua-sz.org (Z.L.); wangs@tsinghua-sz.org (S.W.); 3 School of Mechanical and Manufacturing Engineering, The University of New South Wales, Sydney, NSW 2052, Australia; z.peng@unsw.edu.au

**Keywords:** nanomechanical properties, nanotribology, PEEK, CFRPEEK

## Abstract

This study was to investigate the nanomechanical and nanotribological properties of polyether ether ketone (PEEK)-based composites for biomedical applications and to gain a fundamental understanding of the effects of carbon fibers in carbon-fiber-reinforced PEEK (CFRPEEK) on the mechanical properties and wear performance in a microscale. Nanoindentation tests with a Berkovich indenter and nanoscratch experiments with a diamond stylus were performed on PEEK and CFRPEEK samples. The nanowear features and mechanisms of the tested samples were analyzed using 3D white-light interfering profilometry and scanning electron microscopy (SEM). The obtained results indicated that the reinforced carbon fibers increased the nanohardness and elastic modulus and decreased the friction coefficient and wear rate of PEEK. Different to many existing studies where a constant load was used in a nanoscratch test and the normal load was a key factor influencing the scratch performances of the tested specimens, stick–slip phenomena were observed on both PEEK and CFRPEEK in the nanoscratch tests with load increasing progressively. In constant load conditions, it was found that the major nanowear mechanisms of PEEK are adhesion, abrasion, and plastic deformation, while the nanowear mechanisms of CFRPEEK are dominated by severe adhesive wear, abrasive wear and mild fatigue. CFRPEEK has demonstrated superior nanomechanical and nanotribological performances, and hence can be considered a potential candidate for biomedical applications.

## 1. Introduction

Over the past decades, polyether ether ketone (PEEK) has emerged as a leading high-performance thermoplastic candidate for replacing metal materials in the field of biomedical applications owing to its optimum radiation permeability, biocompatibility, and other advantages [1,2]. PEEK has been used as a substitute for metal components, especially in trauma, orthopedic, and spinal implants to replace diseased or damaged human tissue. With the continuous increase in young and active patients, the recent rise in the use of PEEK in biomedical applications demands further improvement to its anti-wear durability [3]. Existing modifications of PEEK, such as using PEEK as a base polymer matrix in combination with some biocompatible particles [4] or fibers [5], enhance its wear performances. Recently, new developments in carbon-fiber-reinforced PEEK (CFRPEEK) have made it an attractive candidate in biomedical applications because it is reported that carbon fibers can further strengthen the mechanical and wear properties [6]. The conventional tribological properties of PEEK and CFREEK in biomedical applications have been investigated and documented. Díaz et al. [7] compared the tribological performances of ultrahigh molecular weight polyethylene (UHMWPE) and PEEK (against CoCrMo and Al_2_O_3_) using a pin-on-disc contact sliding configuration and indicated that the plasma treated PEEK demonstrates better wear performance than UHMWPE. Xiong et al. [8] also investigated the tribological properties of PEEK, UHMWPE, and PEEK/UHMWPE composites under various lubrication conditions and reported that the friction coefficients and wear rates of PEEK and PEEK/UHMWPE were inferior to those of UHMWPE. Scholes et al. [9] investigated the wear behaviors of pitch and polyacrylonitrile (PAN)-based CFRPEEK articulating against ceramic plates using a pin-on-plate testing machine. The obtained results suggested that the wear rates of pitch and PAN-based CFRPEEK against ceramic are much lower than those of conventional joint materials combinations (metal-on-polyethylene and metal-on-metal) obtained under the same conditions.

Despite implanted biomedical devices being designed for fully lubricated systems, asperity-to-asperity contact occurs at a microscopic scale when lubrication is insufficient to separate two surfaces [10]. It was reported that the real contact area is significantly smaller than the nominal one, leading to much higher contact stress at the bearing surfaces during the wear process [11]. Nanotribology, also called micro-tribology or molecular tribology, focuses on the friction, lubrication, and wear behaviors of interacting surfaces in relative motion at the nanometer scale, which is valuable in understanding the interfacial behaviors in macrostructures to provide a bridge between science and engineering [12]. Wu et al. [13] evaluated the nanoscale tribological performances of the metal asperity-on-UHMWPE interface using atomic force microscopy (AFM) and suggested that plastic deformation and material accumulation take place with an increase in normal loads. Similar work was conducted by Ho et al. [14]. The obtained results indicated that plastic deformation could result in an ultimate failure of the material at the nanotribocontact upon repeated sliding and ploughing.

According to a previous study [15], nanoindentation and nanoscratch tests can be used to detect the material properties and mechanical changes of PEEK composites, especially in the interface/interphase region of the PEEK matrix and reinforced fibers. Molazemhosseini et al. [16] examined the micromechanical properties of PEEK based hybrid composites reinforced with short carbon fibers and nano-SiO_2_ particles using nanoindentation and nanoscratch methods, and showed notable differences between the nanoindentation responses and the microfrictional performances of the PEEK-based composites in matrix and fiber phases. Although the above investigations give good examples to explore the nanomechanical and nanowear characteristics of polymeric biomaterials, further studies on the nanotribological performances for biomedical applications with specific requirements are needed. Compared with conventional tribological experiments carried out at the macroscale, the sliding speed in nanoscratch tests are significant lower [17]. According to previous studies [18,19], an applied normal load of 50–60 mN is widely used for investigating the nanomechanical and nanowear characteristics of biomaterials in nanoscratch tests. It is noted that the stroke of the nanoscratch tests for biomaterials is about 200 μm [20]. Hence, investigations under those specific conditions are required to further understand the nanofriction and nanowear properties of PEEK and CFRPEEK for biomedical applications (e.g., artificial joints).

This work was motivated by such considerations. The nanomechanical and nanotribological behaviors of PEEK and CFRPEEK for biomedical applications were quantitatively investigated through nanoindentation and nanoscratch experiments. The nanowear features and mechanisms were analyzed by examining the post-test worn surfaces. A better understanding of the asperity-to-asperity contact behaviors of PEEK and CFRPEEK will be achieved.

## 2. Experimental

### 2.1. Materials and Sample Preparation

In this study, disc-shaped samples (with a diameter of 20 mm and a thickness of 5 mm) were made from neat PEEK (Victrex PEEK™ grades 450G) and CFRPEEK (Victrex PEEK™ grades 450CA30, namely the PEEK™ grades 450G reinforced by 30 wt % random-oriented short carbon fibers derived from polyacrylonitrile), respectively. The neat PEEK was used for the purposes of comparison. The specimens were successively ground using 60 to 1500 mesh Al_2_O_3_ abrasive papers before final polishing with diamond paste to achieve a mirror-like surface finish and an average surface roughness (Ra) 0.10 ± 0.02 μm, measured by a 3D white-light interfering profilometer (Nexview, Zygo, Middlefield, OH, USA).

### 2.2. Nanoindentation and Nanoscratch Tests

In order to study the nanomechanical properties of PEEK and CFRPEEK, the nanoindentation tests were conducted on a nanomechanical tester (NHT^2^, CSM Instruments SA, Peseux, Switzerland) at a peak load of 3 mN and a loading and unloading rate of 6 mN/min with a Berkovich indenter. The mean values of nanohardness (H) and reduced elastic modulus ($E_{r}$) were obtained by averaging at least three different surface positions for each specimen. 

Nanoscratch tests were performed on the same tester with a diamond stylus in a radius of 2 μm to investigate the nanotribological properties of the PEEK and CFRPEEK samples [21]. Progressive and constant load nanoscratch tests were carried out for each sample, respectively. The progressive load nanoscratch tests were performed with an initial load of 0.3 mN and a final load of 60 mN as 3-scan (topography-progressive load scratch-topography) experiment to investigate the nanowear performances of the PEEK and CFRPEEK specimens. In order to further study the influence of the applied normal load on the scratch performances, the constant load nanoscratch experiments were carried out as multi-pass reciprocating nanowear tests (topography-10 constant load scratches-topography) under 10, 30, and 60 mN, respectively. The time span for one scratch stroke was set up as 1 min to avoid the viscoelastic effect of PEEK and CFRPEEK [22]. The tests were conducted in dry conditions to simulate the asperity-to-asperity contact in severe conditions when a lack of lubrication occurred. The friction force and on-load scratch depth values were acquired using sensors simultaneously. The friction coefficient was obtained by dividing the frictional force by the normal force. The main parameters in the nanoscratch tests are displayed in Table 1.

### 2.3. Post-Test Analysis

After the nanoscratch tests, the worn surfaces of PEEK and CFRPEEK samples were analyzed using a 3D white-light interfering profilometer (Nexview, Zygo, Middlefield, OH, USA). The surface profile as well as the wear widths and depths of the wear tracks were calculated using Zygo’s MetroPro software (Berwyn, PA, USA). In order to further understand the nanowear features and mechanisms, the 2D subsurface damages were investigated in this study. High-resolution images of the worn surfaces of the PEEK and CFRPEEK specimens were imaged using scanning electron microscopy (SEM, FEI Quanta 200 FEG, Eindhoven, The Netherlands). Before the SEM observation, the samples were ultrasonic cleaned, and then sputter-coated with a thin carbon layer to improve image quality. All quantitative results were obtained by averaging the values of three repetitions.

One-way analysis of variance and Least-Significant Difference (LSD) post hoc tests were carried out in SPSS^®^ v.20.0.0 (SPSS Inc., Chicago, IL, USA) to evaluate the differences of the results obtained in this study. The significance level was established at *p* < 0.05.

## 3. Results

### 3.1. Nanoindentation

**Figure 1:**
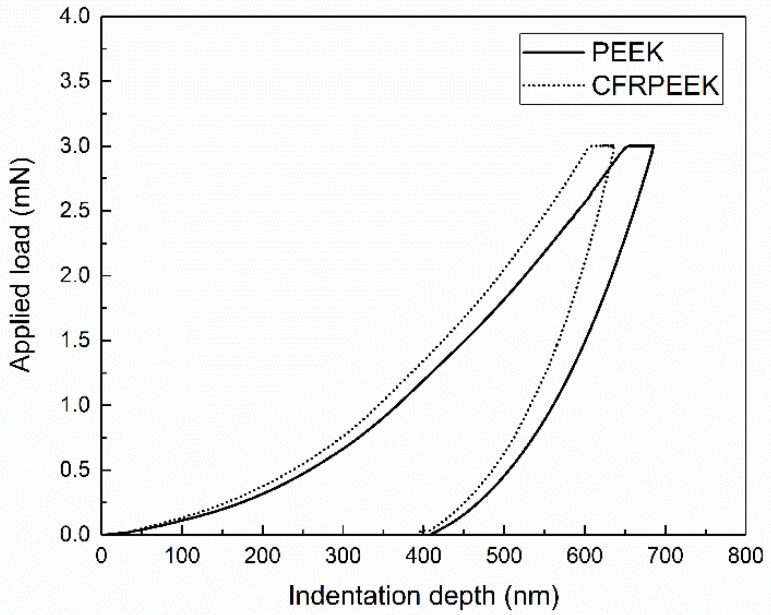
Typical nanoindentation curves of PEEK and CFRPEEK at a peak load of 3 mN with a Berkovich indenter.

The load-displacement curves for the nanoindentation tests on PEEK and CFRPEEK with a peak load of 3 mN are presented in Figure 1. The values of the indentation depth of PEEK and CFRPEEK at the maximum applied load (3 mN) increase with the loading time, revealing the creep effect of the tested PEEK and CFRPEEK. More specifically, the values of nanohardness (H), reduced elastic modulus ($E_{r}$) and their ratio ($H/E_{r}$) obtained in the nanoindentation tests are displayed in Table 2. The nanohardness and the reduced elastic modulus of CFRPEEK were higher than those of PEEK, indicating that the reinforced carbon fibers improved the mechanical properties of PEEK, which is similar to previous literature [23]. Because of the reinforced carbon fibers, the obtained results in CFRPEEK had a larger standard deviation than those of the PEEK samples. According to the statistical analysis, there is a significant difference between the $E_{r}$ values of PEEK and CFRPEEK. Table 2 also suggests that the $H/E_{r}$ ratio of PEEK is higher in comparison to that of CFRPEEK. It is reported that the higher the $H/E_{r}$ ratio, the higher the indentation recovery [10]. PEEK demonstrates a higher indentation recovery. A further discussion of the nanoindentation performances of PEEK and CFRPEEK can be found in Section 4.

### 3.2. Nanofriction Properties

**Figure 2:**
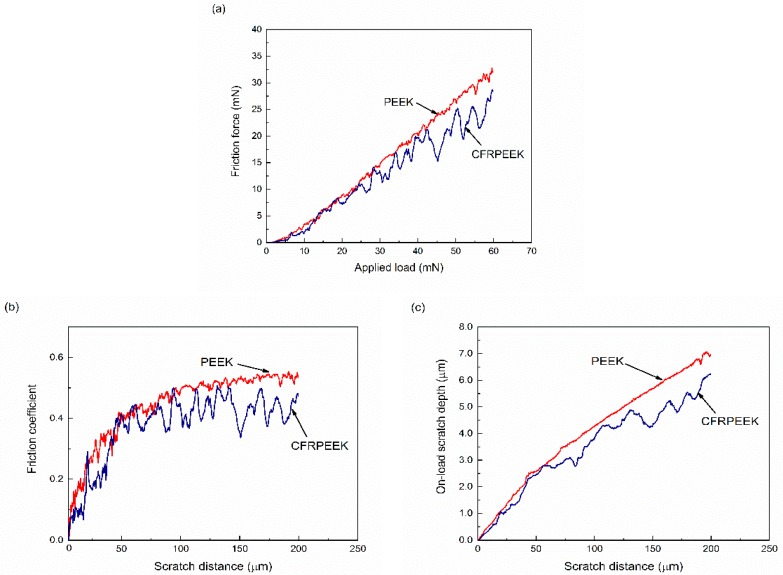
Variations of (**a**) friction force and (**b**) friction coefficient and (**c**) on-load scratch depth of PEEK and CFRPEEK in progressive load nanoscratch tests with an initial load of 0.3 mN and a final load of 60 mN.

In order to have an insight into the nanofriction behaviors of PEEK and CFRPEEK, their friction forces, friction coefficients, and on-load scratch depths in the progressive nanoscratch tests are displayed in Figure 2. Figure 2a shows that the values of the friction forces increased with applied load, indicating a higher and smoother friction force of PEEK in comparison to that of CFRPEEK. In addition, CFRPEEK had a similar friction force with PEEK at the beginning of the tests. Owing to the reinforced carbon fibers in the PEEK matrix, the friction force of CFRPEEK fluctuated severely when the applied load was above 30 mN. As displayed in Figure 2b, the friction coefficients of the tested specimens increased sharply at the initial stage, and then fluctuated in a stabilized range. The average friction coefficient of CFRPEEK in the progressive load nanoscratch test was approximately 0.362, which is 84.5% of the mean friction coefficient of PEEK, indicating better tribological performance of CFRPEEK. Figure 2c shows that the on-load scratch depth of CFRPEEK almost increased linearly at the beginning of the tests with relative low load (<30 mN), which is similar to PEEK. With the increasing scratch distance, the on-load scratch depth curve of CFRPEEK began to fluctuate, indicating that the reinforced carbon fibers played the roles to support the applied load and prevent the macromolecules in the composite matrix from removing easily [24]. In addition, the statistical analysis indicates that the obtained friction forces, friction coefficients and on-load scratch depths of PEEK were all significantly different with those of CFRPEEK (*p <* 0.05).

**Figure 3:**
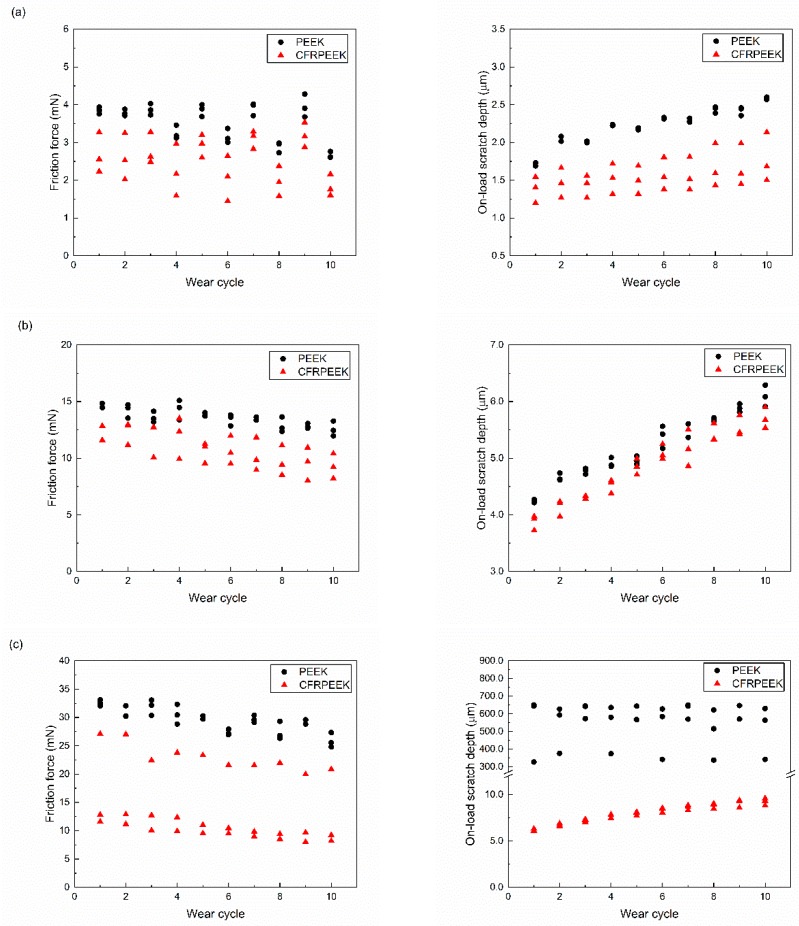
The evolution of friction force (left) and on-load wear depth (right) of PEEK and CFRPEEK under different applied load: (**a**) 10 mN, (**b**) 30 mN, and (**c**) 60 mN.

**Figure 4:**
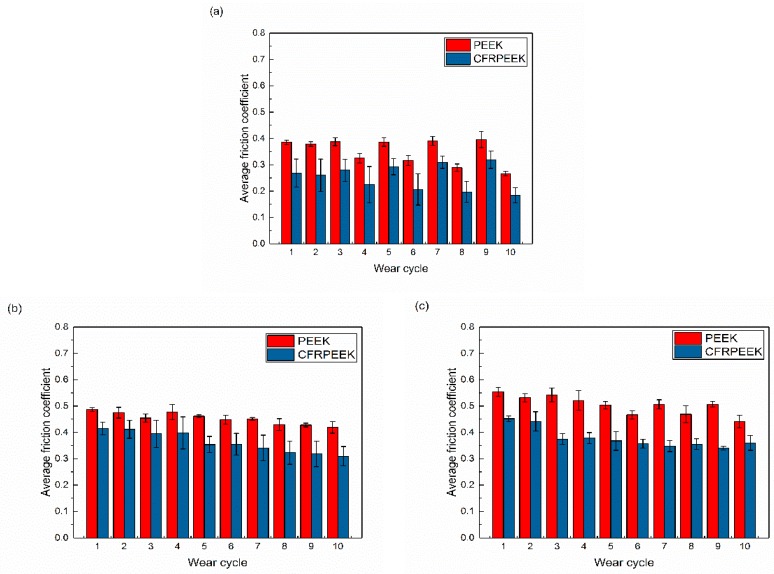
The average friction coefficients of PEEK and CFRPEEK in the constant load nanoscratch tests: (**a**) 10 mN, (**b**) 30 mN, and (**c**) 60 mN.

The constant load nanoscratch tests were performed to further study the nanotribological behaviors under different loading conditions and the obtained results are displayed in Figure 3. The friction forces of the tested specimens decreased and the probe depths under load increased with each successive wear scan. As can be seen in Figure 3, the friction forces and on-load scratch depths of CFRPEEK were lower than those of PEEK, which is in agreement with the results obtained in progressive load scratch tests (Figure 2). It is indicated that the gap of the obtained results between the PEEK and CFRPEEK under the applied load of 60 mN was higher than in the other two conditions, especially for the on-load probe depth. It was reported that the increase in the modulus has a positive effect on the anti-wear characteristics of PEEK composites [25]. A higher modulus suggests a lower wear rate generally. Hence, it was expected that the on-load scratch depth of CFRPEEK was much lower than that of PEEK, owing to the higher modulus shown in Table 2. The average friction coefficients of PEEK and CFRPEEK in the constant load nanoscratch tests are shown in Figure 4, which illustrate the dynamic friction between the tested specimens and diamond stylus. The obtained data indicate that the friction coefficients of the tested samples increased with a normal load increment. The friction coefficients of CFRPEEK were also lower than those of PEEK under the same testing condition. More specifically, the friction coefficients obtained in this study were in a range of 0.2–0.5, which are similar to our previous studies in a macrometer scale [26,27]. To have a better understanding of the nanotribological behaviors of PEEK and CFRPEEK, in addition to the friction coefficient, the nanowear features and mechanisms should be investigated (see Section 3.3).

### 3.3. Nanowear Features and Mechanisms

**Figure 5:**
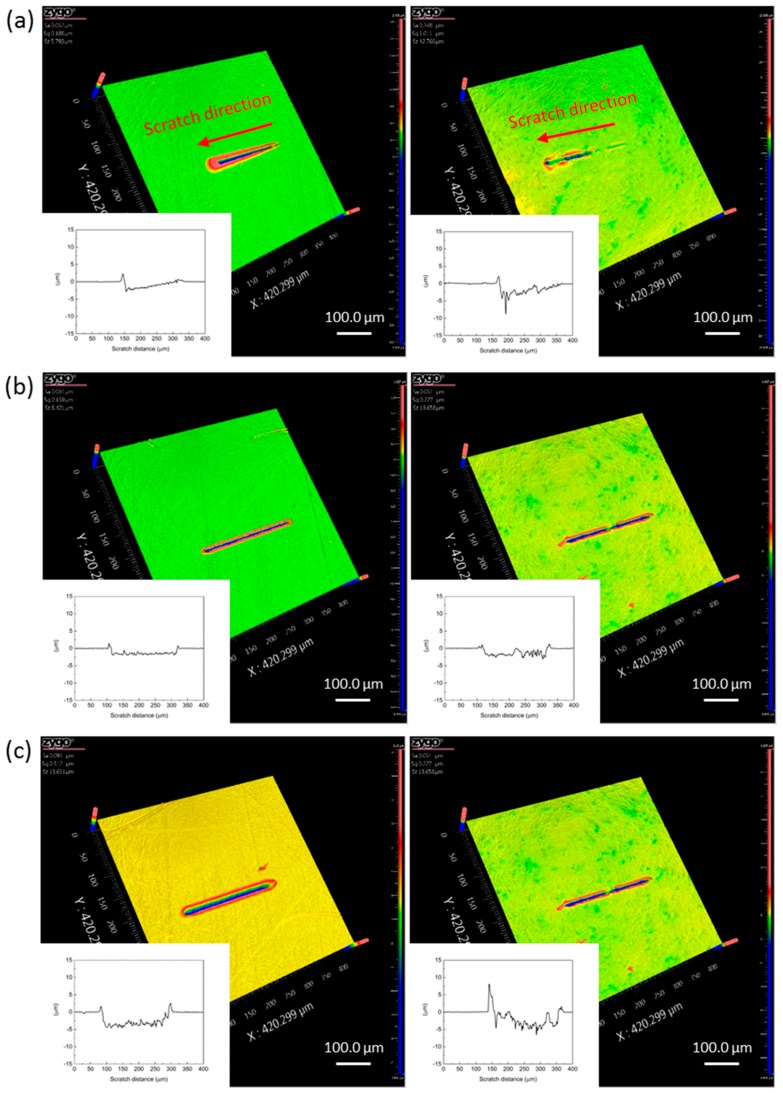

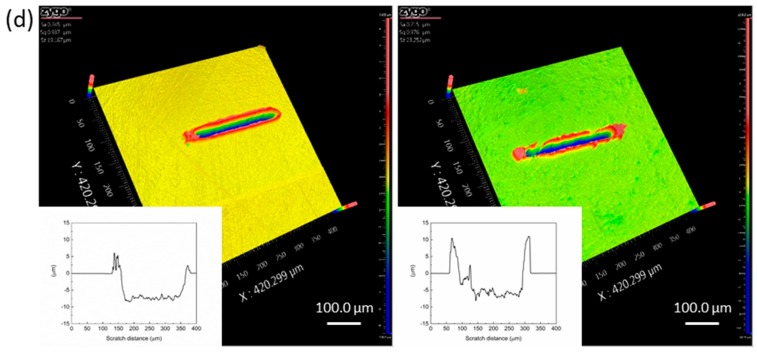
3D profile micrographs of the worn surfaces on the PEEK (left) and CFRPEEK (right) discs at different applied load: (**a**) progressive load with an initial load of 0.3 mN and a final load of 60 mN, (**b**) constant load of 10 mN, (**c**) constant load of 30 mN, and (**d**) constant load of 60 mN. The midline profile of each wear track in the sliding direction was displayed in the left bottom.

To have a better understanding of the nanotribological properties of PEEK and CFRPEEK, 3D worn surfaces of the tested samples in the progressive and constant load conditions are presented in Figure 5. It can be seen that the wear tracks of the tested specimens varied with different experimental parameters. Figure 5a shows that the wear tracks of PEEK and CFRPEEK in the progressive load nanoscratch tests were needle-shaped because of the load increase. The wear track on CFRPEEK was not obvious at the beginning of the test, but the wear width and depth increased with the increasing applied load. These wear features reveal that the CFRPEEK had better wear resistance than PEEK, which is in agreement with the data presented in Figure 3. In terms of the constant load conditions, the wear tracks are all narrow scratch grooves surrounded by heaps of materials (pile-up) displaced under the plowing effect. Figure 5b–d also indicate a rise in the wear widths and depths of PEEK and CFRPEEK samples with the increasing normal load.

To quantify the nanowear behaviors of the tested specimens, the mean values of the wear width, wear depth, and pile-up height of the nanowear tracks at different constant normal loads were obtained and are presented in Table 3. The wear rates of the tested samples increased with the increasing normal load, which is in conformity with the results presented in Figure 5. The wear width and depth of CFRPEEK were lower in high load (≥30 mN) conditions than those of neat PEEK, indicating the improvement of the nanowear resistance of CFRPEEK. The statistical analysis indicates that the differences between the wear depths of PEEK and CFRPEEK are all significant. More specifically, the average wear width and wear depth of CFRPEEK at the applied load of 60 mN were 93.8% and 72.9% of those of PEEK, respectively. As displayed in Table 3, in comparison with PEEK, although the wear rates of CFRPEEK were lower, the pile-up heights were higher, indicating the wear debris of CFRPEEK on the worn surface was cumulated easily.

**Figure 6:**
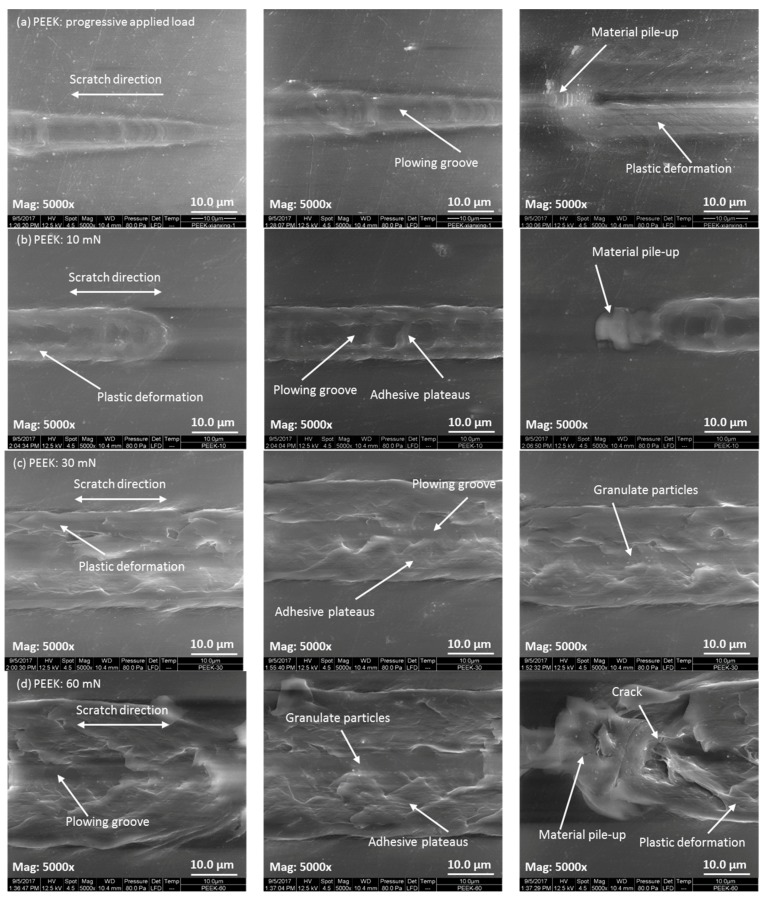
The morphologies of the worn surfaces observed by SEM for PEEK with different applied loads: (**a**) progressive load with an initial load of 0.3 mN and a final load of 60 mN; (**b**) constant load of 10 mN; (**c**) constant load of 30 mN; and (**d**) constant load of 60 mN. The beginning, middle, and end of the wear scar are displayed in the left, middle, and right columns, respectively.

**Figure 7:**
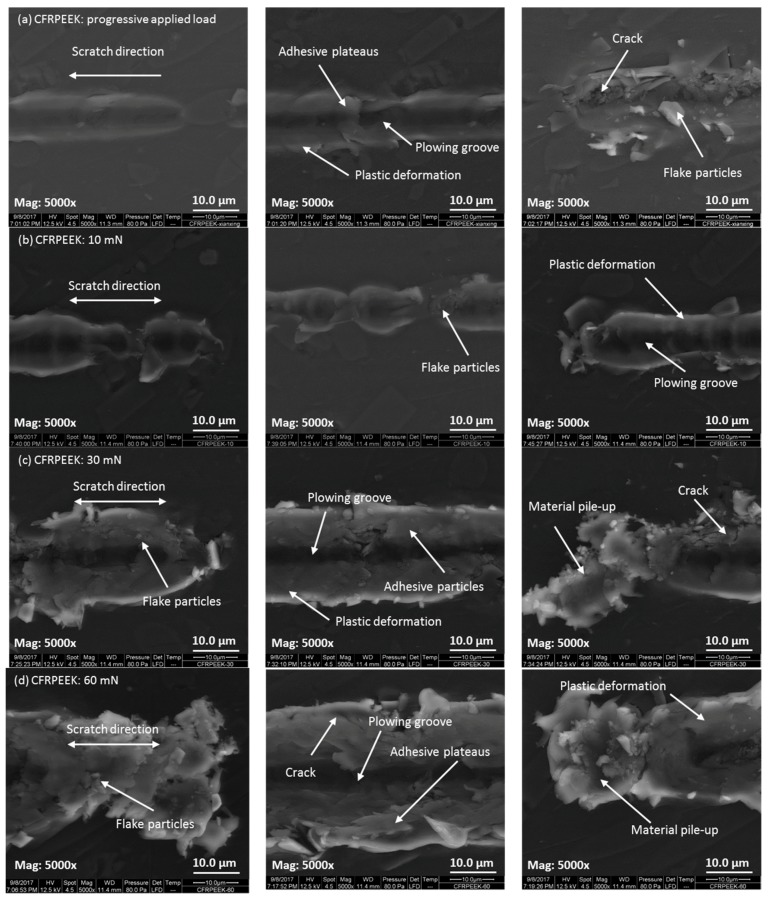
The morphologies of the worn surfaces observed by SEM for CFRPEEK with different applied loads: (**a**) progressive load with an initial load of 0.3 mN and a final load of 60 mN; (**b**) constant load of 10 mN; (**c**) constant load of 30 mN; and (**d**) constant load of 60 mN. The beginning, middle, and end of the wear scar are displayed in the left, middle, and right columns, respectively.

In order to explore the nanowear features and mechanisms, the SEM micrographs of the worn surfaces of PEEK and CFRPEEK were acquired and are displayed in Figure 6 and Figure 7, respectively. It is noted that the micrographs of the post-test worn surfaces on PEEK and CFRPEEK are different. Under the progressive applied load condition, obvious plowing grooves were found in the central region of the wear tracks of PEEK and CFRPEEK because of the scratch of the hard diamond stylus. The widths of the wear tracks increased with the increasing normal load, showing the needle-like shape on the worn surfaces. It is suggested that the deformation produced in the tests is elastic at low applied force and converted into plastic under high normal force. Compared with CFRPEEK, there was severe plastic deformation on the worn surface of PEEK. In addition, a large quantity of wear cracks, as well as flake-like and adhered wear debris, were observed in Figure 7a, which are different from the features of PEEK shown in Figure 6a. Thus, the major nanowear mechanisms of PEEK under progressive applied load are abrasive wear and severe plastic deformation. The nanowear mechanisms of CFRPEEK are mainly abrasive and adhesive wear plus mild plastic deformation. 

In terms of the constant load conditions, the features of plastic deformation, including the materials piling up around the plowing grooves, were observed in the high magnification (Mag: 5000×) SEM images of the wear tracks of PEEK and CFRPEEK. The degree of the materials piling up increased with the increasing normal load, suggesting the increased plastic deformation and wear rate with load. This trend is in agreement with the obtained data shown in Table 3. It is noted that there were some cracks, granulate wear debris and adhesive plateaus on the worn surfaces of PEEK as shown in Figure 6b–d, indicating that the nanowear mechanisms of PEEK under constant loads are dominated by adhesive wear, abrasive wear and plastic deformation. Compared with PEEK, more wear debris were observed on the worn surfaces of CFRPEEK, which were evenly adhered around the scratch grooves. In addition, Figure 7b also indicates a discontinuous wear track at low load (10 mN), which is one reason why the wear track on CFRPEEK was not obvious at the beginning of the progressive load nanoscratch test. Thus, the major nanowear mechanisms of CFRPEEK are severe adhesion, abrasion and mild fatigue. Further discussion on the correlation between the nanofriction and nanowear performances of the tested specimens can be found in the following section.

## 4. Discussion

Recently investigations on the tribological characteristics of biomedical polymers are being transferred from the macroscale to the microscale [13], which is of importance for developing an understanding of the interfacial phenomena of the tested samples in a small scale. From the biomedical applications (e.g., artificial joints) point of view, multiple asperity contacts occur during the sliding between two surfaces articulated against each other, leading to the microscale contact of the components in artificial joints. 

Nanoindentation is an appropriate approach for evaluating the hardness and elastic modulus profiles in a micron or submicron range [28]. According to the results obtained, the reinforced carbon fibers improved the nanomechanical performances of PEEK. It was reported that reinforced carbon fibers have a significant influence on the morphological properties and the polymer chain alignment of PEEK when they are in contact with a solid surface during solidifying [15], leading to a higher nanohardness value in comparison with neat PEEK. As can be seen in Table 2, the reduced elastic modulus of CFRPEEK (8.481 GPa) demonstrates an increase of 35% in comparison with the one of PEEK (6.260 GPa). Furthermore, Figure 3 indicates that there is a huge gap between the on-load probe depths of PEEK and CFRPEEK at the normal load of 60 mN. Although the statistical analysis indicates the difference between the post-test wear depths of PEEK and CFRPEEK presented in Table 3 is significant, the gap between the values is much smaller than the on-load probe depths. It is suggested that those induced carbon fibers restricted the molecular movement and decreased the degree of normal plastic-flow deformation of the PEEK matrix [29]. As a result, a higher reduced elastic modulus of CFRPEEK was measured, and severe, localized material pile-ups were observed on the wear tracks of CFRPEEK.

The obtained results in this study suggest that the nanotribological performances of CFRPEEK under the progressive load condition were improved in comparison to those of PEEK. The friction coefficient curve of PEEK and CFRPEEK presented in Figure 2b periodically increased and decreased, indicating the stick–slip motion between the indenter tip and surfaces of PEEK and CFRPEEK during the tests [30]. More specifically, for CFRPEEK, only small stick–slip fluctuations were found with low applied load (<30 mN). When the normal load was over 30 mN, the fluctuation magnitude of the friction coefficient curve became larger. Compared with the conventional tribological evaluations for biomaterials, the applied load in this study is much lower (a peak load of 60 mN). It is reported that the stick–slip motion, a typical friction-induced vibration, can be observed at low sliding speeds when an indenter is driven through an elastic material [31]. In the stick stage, although the indenter tip and the surfaces of PEEK (or CFRPEEK) have almost zero relative motion, the stress yielded by the normal load is still on the specimen surface, causing the deformation of the material. Once the applied stress exceeds the required critical stress for relative motion, the slip stage initiates, resulting in the plastic deformation and accumulation of the material in front of the indenter tip [32]. Moreover, the optimum friction and wear properties of CFRPEEK at constant load conditions can be attributed to the reinforcement of carbon fibers. It is suggested that the tribological behaviors of the composites are dominated by carbon fibers, which possess high clash modulus and strength, determining the load action on the composites [33].

Figure 6 and Figure 7 indicate that the nanowear features of PEEK and CFRPEEK varied with the applied load, suggesting that the normal load was a key factor affecting the scratch performances of PEEK and CFRPEEK. As can be seen in Figure 7, a large amount of wear debris were seen on the worn surfaces of CFRPEEK in different load conditions, leading to higher pile-ups than on neat PEEK. The material pile-up effect was severe at the end of the scratch, which is similar to the results obtained in a previous study [13]. Larger pile-up height is closely related to higher contact pressure and shear force, indicating easier formation of the wear debris. Pei et al. [21] suggested the sequence of the damaging mechanisms of polymers under the effects of single asperity geometry is: plowing, fracture in the groove, formation of patchy layers, as well as stretching and tearing of deformed layers, which is in agreement with the nanowear features of PEEK observed by SEM in this study. As mentioned above, the reinforced carbon fibers in PEEK matrix can support the normal load and restrict the removal of the macromolecules. As a result, few patchy layers (adhesive plateaus) were observed in Figure 7. In addition, the wear debris of CFRPEEK obtained in this study are mainly in the microscale, as shown in Figure 7. According to a previous study [34], micro-sized debris might induce less DNA damage, aneuploidy, and cytotoxicity in comparison with nano-sized. It is reported that CFRPEEK generated a significant lower debris weight than the conventional metal alternatives in biomedical applications [35]. Furthermore, available investigations strongly support CFRPEEK as a suitable candidate for biomedical applications because of its biocompatibility [36]. Thus, from the biocompatibility point of view, we assume that the micron-sized wear debris of CFRPEEK obtained in this study may not result in immunological reaction, indicating that CFRPPEK can be a potential candidate for biomedical applications.

However, more investigations are required to comprehensively evaluate the clinical use of CFRPEEK. Further studies will be conducted using specific tips modified by biomaterials in lubricated condition to simulate the real material combinations of biomedical devices (e.g., artificial joints). Post-test wear particles will be collected and studied to further understand nanowear mechanisms and biocompatibility. 

## 5. Conclusions

The nanomechanical and nanotribological behaviors of PEEK and CFRPEEK were studied using a Berkovich indenter in a nanoindentation mode and a diamond stylus with the radius of 2 μm in a nanoscratch mode. The main conclusions drawn are as follows:

(1) The induced carbon fibers in PEEK matrix increased the nanohardness and reduced elastic modulus, which have a beneficial effect on the nanotribological performances of PEEK.
(2) The friction coefficient and wear rate of CFRPEEK was lower than that of PEEK. However, a higher material pile-up of CFRPEEK was found, which was closely related to higher contact pressure and shear force, leading to easier formation of the wear debris.
(3) When tested in the progressive load nanoscratch mode, the major nanowear mechanisms of PEEK were abrasive wear and severe plastic deformation, and those of CFRPEEK were mainly abrasion, adhesion, and mild plastic deformation.
(4) The normal load had a significant influence on the scratch performances in the constant load nanoscratch tests. The major nanowear mechanisms of PEEK were adhesive wear, abrasive wear, and plastic deformation. The nanowear mechanisms of CFRPEEK were dominated by severe adhesive wear, abrasion, and mild fatigue wear.
(5) CFRPEEK has demonstrated superior nanomechanical and nanotribological performances in this study, and hence is a potential candidate for biomedical applications.

## Figures and Tables

**Table 1:** Experimental parameters in the nanoscratch tests using the 2 μm probe.

	Progressive Load Nanoscratch	Constant Load Nanoscratch
Tangential movement	Linear	Reciprocating
Track lengh (μm)	200	200
Scan speed (μm/s)	5	5
Applied load (mN)	0.3–60	10, 30, 60
Loading rate (mN/min)	90	-
Hold at peak load (s)	-	60 (per cycle)
Total scratch cycles	1	10

**Table 2:** Values of nanohardness (H), reduced elastic modulus ($E_{r}$) and their ratio ($H/E_{r}$, $H^{3}/E_{r}^{2}$) obtained in the nanoindentation tests. * *p* < 0.05, statistically significant difference; PEEK versus CFRPEEK.

	H (GPa)	$E_{r}$ (GPa)	$H/E_{r}$
PEEK	0.326 ± 0.018	6.260 ± 0.405	0.052
CFRPEEK	0.345 ± 0.033	8.481 ± 1.507 *	0.041

**Table 3:** Measurement of nanowear tracks for PEEK and CFRPEEK after the nanoscratch tests. * *p* < 0.05, statistically significant difference, PEEK versus CFRPEEK in the same experimental conditions.

	Applied Load (mN)	Wear Width (μm)	Wear Depth (μm)	Pile-up Height (μm)
PEEK	10	10.103 ± 0.301	1.392 ± 0.567	1.521 ± 0.101
	30	19.387 ± 0.526	3.264 ± 0.844	3.631 ± 0.186
	60	28.433 ± 0.325	6.932 ± 1.328	6.018 ± 0.345
CFRPEEK	10	10.107 ± 0.617	1.551 ± 0.658 *	1.796 ± 0.156 *
	30	19.287 ± 0.767	2.731 ± 1.495 *	3.912 ± 0.303
	60	26.692 ± 0.531 *	5.051 ± 1.878 *	6.317 ± 1.165
